# Effects of back school-based intervention on non-specific neck pain in adults: a randomized controlled trial

**DOI:** 10.1186/s13102-023-00666-8

**Published:** 2023-04-17

**Authors:** Pablo Hernandez-Lucas, Raquel Leirós-Rodríguez, Juan Lopez-Barreiro, José L. García-Soidán

**Affiliations:** 1 Faculty of Physiotherapy, University of Vigo, Campus A Xunqueira, Pontevedra, 36005 Spain; 2 SALBIS Research Group, Nursing and Physical Therapy Department, University of Leon, Astorga Ave, Ponferrada, 24401 Spain; 3 Faculty of Education and Sport Sciences, University of Vigo, Campus A Xunqueira, Pontevedra, 36005 Spain

**Keywords:** Exercise therapy, Health education, Neck pain, Musculoskeletal pain, Physical therapy modalities, Rehabilitation

## Abstract

**Background:**

Neck pain has a high prevalence and socioeconomic impact worldwide. The Back School consists of programs that include exercises and educational interventions to treat back pain. Accordingly, the main objective was to evaluate the effects of an intervention based on Back School on non-specific neck pain in an adult population. The secondary objectives were to analyze the effects on disability, quality of life and kinesiophobia.

**Methods:**

A randomized controlled trial was conducted with 58 participants with non-specific neck pain divided into two groups. The experimental group (EG) carried out the 8-week programme based on the Back School, (two sessions per week, for a total of 16 sessions, lasting 45 min). Of all the classes, 14 had a practical focus (strengthening and flexibility exercises) and the other two had a theoretical focus (concepts of anatomy and healthy lifestyle). The control group (CG) stated that they did not vary their lifestyle. The assessment instruments were: Visual Analogue Scale, Neck Disability Index, Short-Form Health Survey-36 and Tampa Scale of Kinesiophobia.

**Results:**

The EG reduced pain (-40 points, CI95% [-42 to -37], g = -1.03, *p* < 0.001), EG had less disability (-9.3 points, CI95% [-10.8 to -7.8], g = -1.22, *p* < 0.001), EG improved the physical dimension of the survey Short-Form Health Survey-36 (4.8 points, CI95% [4.1 to 5.5], g = 0.55, *p* = 0.01) but had not significant change in psychosocial dimension of the survey Short-Form Health Survey-36 and EG reduced Kinesiophobia (-10.8 points, CI95% [-12.3 to -9.3], g = -1.84, *p* < 0.001). The CG did not obtain significant results in any variable of the study. Significant differences in change between both groups were found on pain (-11 points, CI95% [5.6 to 16.6], *p* < 0.001, g = 1.04), disability (-4 points, CI95% [2.5 to 6.2], *p* < 0.001, g = 1.23), physical dimension of the survey Short-Form Health Survey-36 (3 points, CI95% [-4-4 to -2-5], *p* = 0.01, g = -1.88), and kinesiophobia ( 7 points, CI95%[-8.3 to -5.4], *p* < 0.001, g = 2.04), while no significant differences were found on psychosocial dimension of the survey Short-Form Health Survey-36 (-0.02, CI95% [-1.7 to 1.8], g = 0.01, *p* = 0.98).

**Conclusions:**

The back school-based programme has beneficial effects on pain, neck disability, the physical dimension of quality of life and kinesiophobia in an adult population with non-specific neck pain. However, it did not lead to improvements in the psychosocial dimension of the participants’ quality of life. This programme could be applied by health care providers with the aim of reducing the severe socio-economic impact of non-specific neck pain worldwide.

**Trial registration in ClinicalTrials.gov:**

NCT05244876 (registered prospectively, date of registration: 17/02/2022).

**Supplementary Information:**

The online version contains supplementary material available at 10.1186/s13102-023-00666-8.

## Background

Neck pain is one of the most common musculoskeletal disorders worldwide, with an age-standardized prevalence rate of 2.7% in 2019 [1]. It is also a major disease burden in terms of global years lived with disability [2]. The economic consequences of neck pain are significant for both individuals themselves and society due to costs related to healthcare, insurance, lost productivity, and work-related sick leaves [2, 3].

Most neck musculoskeletal disorders do not have an identifiable underlying disease or abnormal anatomical structure, and are therefore classified as Non-specific neck pain (NNP) [4]. It is essential to know the main risk factors associated with NNP in order to act on them, with the aim of reducing the serious socioeconomic repercussions caused by NNP [5]. This disease is multifactorial [1, 6]: sedentary lifestyle [7], lack of strength of the cervical musculature [8–10], psychosocial factors [11, 12] and occupational factors [13–15].

The main clinical practice guidelines include a multimodal approach for the treatment of non-specific back pain with exercises, advice and education [6]. One of the most widely used non-pharmacological tools in the treatment of back pain is the Back School Program (BSP), initiated in Sweden in 1969 by physiotherapist Zachrisson Forssell [16]. BSP consiste en un.

programa teórico-práctico que pretende enseñar habilidades que protejan la salud de la espalda a personas sanas o con patología de espalda [16].

Currently, back pain treatment programmes follow the biopsychosocial model of pain [17]. Consequently, the new BSPs take this into account by conveying in their theoretical part healthy lifestyle recommendations and information about erroneous beliefs about the causes of NNP. In addition, in the practical part, patients are taught how to perform back strengthening and stretching exercises [18–20]. There is scientific evidence on the beneficial effects of BSP in people with low back pain: improvement of quality of life [21–23], reduction of pain [21–26], prevention of pain [20] and reduction of disability [21–27]. However, there is little evidence on these effects of BSP on the cervical region [28]. A previous review concluded that the quality of published studies on BSP is of low methodological quality and that there is a need for further research analysing new BSP variants [29]. Therefore, the main objective of this study was to evaluate the effects of an intervention based on BSP on non-specific cervical pain in an adult population with NNP. The secondary objective was to analyze the effects of the BSP intervention on disability, quality of life and kinesiophobia, with the hypothesis that BSP has positive effects on decreasing pain, decreasing disability, improving quality of life and decreasing kinesiophobia in adults with NNP compared to patients with the same pathology who did not perform BSP.

## Methods

### Study design

A randomized controlled clinical trial was conducted, in which scores on measures of the dependent variables were compared before and after the intervention, both in the experimental group (EG) (people who attended the BSP) and in the control group (CG) (people who did not attend the BSP). The experimental procedure followed the CONSORT and TidIER guidelines. The study protocol was approved by the University of León Research Ethics Committee (code: ULE-013-2022), registered on ClinicalTrials.gov (NCT05244876), and this study was conducted under the Declaration of Helsinki (2013 version). After being informed of the benefits and risks of research, participants signed written informed consent.

### Participants

Participants were recruited on a voluntary basis and without any financial remuneration. To recruit participants, information posters were put up in the Pontevedra Sport Center (Spain). Seventy-six volunteers presented with the following inclusion criteria: (i) age between 18 and 65 years; (ii) non-specific neck pain for at least three months, with pain intensity of 30–70 on the Visual Analogue Scale (VAS). The following exclusion criteria were also applied: (i) having no previous neck or shoulder surgery, medical diagnosis of fibromyalgia, cervical radiculopathy/myelopathy, history of whiplash injury, or cognitive disorder. They were randomly divided into two 1:1 group; the assignment was concealed by sealed opaque envelopes.

### Intervention

The intervention consisted of a program based on BSP. This program followed the recommendations of the biopsychosocial model of chronic pain [17]. The intervention was carried out within the physiotherapy area of a sports center. The duration of the intervention was eight weeks with a frequency of two sessions per week, making a total of 16 sessions lasting 45 min. Of all the sessions, 14 had a practical focus and the other two had a theoretical focus. All participants were informed of the importance of attending the sessions and attendance was monitored. A summary of the intervention and procedure carried out in this study is shown in Table 1.

- Theoretical sessions: The theoretical sessions were given by a registered physiotherapist. During the first 30 min, the physiotherapist gave a presentation with the help of videos and anatomical models. The following 15 min, a group discussion was held, and the participants’ doubts were answered. In the first theoretical session, basic concepts of biomechanics were explained and misconceptions about NNP were clarified. In the second theoretical session, the main psychosocial factors of NNP were explained. The theoretical sessions were conducted face-to-face and in groups of maximum 10 participants.
- Practical sessions: The practical sessions were given by a registered physiotherapist. They had the following structure: doubts, warm-up, main part, and cool-down. The first part of each practical session lasted approximately three minutes, during which the participants asked questions and reviewed the basic principles of each exercise. In the sessions in which there were no doubts, the physiotherapist took the opportunity to ask questions about the content seen in the theoretical classes with the aim of recalling knowledge, thus integrating both parts: theory and practice. The warm-up lasted seven minutes, during which joint mobility exercises were performed. The main part lasted 30 min. In this part, strength and endurance exercises of the cervical and scapular region, using an elastic band, were alternated with active breaks consisting of soft joint mobility exercises. Every three sessions, a 25% increase was applied to the pull-force by varying the resistance of the elastic band (Appendix 1). The cool-down lasted five minutes, during which the focus was on flexibility, breathing and relaxation exercises. The practical sessions were held in groups of a maximum of 10 participants.

**Table 1:** Summary of the intervention and procedure

Session number	Session type	Name	Main objective of the session
1	Theoretical.	Biomechanics and risk factors.	Learning the basics of biomechanics, risk factors and clarification of erroneous beliefs regarding the causes or origin of non-specific neck pain.
2–4	Practical.	Exercises with thin resistance band.	Doing and learning strength and endurance exercises with thin resistance band. 1.3 kg resistance at 100% elongation.
5	Theoretical.	Psychosocial factors of the non-specific neck pain.	Knowing psychosocial factors of the non-specific neck pain.
6–8	Practical.	Exercises with medium resistance band.	Doing and learning strength and endurance exercises with medium resistance band. 1.7 kg resistance at 100% elongation.
9–11	Practical.	Exercises with heavy resistance band.	Doing and learning strength and endurance exercises with heavy resistance band. 2.1 kg resistance at 100% elongation.
12–14	Practical.	Exercises with extra heavy resistance band.	Doing and learning strength and endurance exercises with extra heavy resistance band. 2.6 kg resistance at 100% elongation.
15–16	Practical.	Exercises with special heavy resistance band.	Doing and learning strength and endurance exercises with special heavy resistance band. 3.3 kg resistance at 100% elongation.

### Variables analysed

Two evaluation sessions were conducted at the beginning and at the end of the intervention, in which sociodemographic and anthropometric data were collected: age, sex, weight (using a Tanita™ b303 scale, Tokyo, Japan), and height (using a homologated Seca™ 709 height rod, Hamburg, Germany).

#### Pain intensity

The VAS tool is widely used to measure pain. The patient is asked to indicate his/her perceived pain intensity (most commonly in the last 24 h) along a 100 mm horizontal line. The left edge shows the absence and the right edge shows the highest intensity of pain [30].

#### Disability

The Neck Disability Index (NDI) was the test used to measure disability, as it is the most strongly validated instrument for assessing self-rated disability in patients with neck pain. The questionnaire has 10 items including pain, self-care, lifting, reading, headaches, concentration, work, driving, sleep, and leisure. The score interpretation for the NDI is: 0–4 = no disability; 5–14 = mild disability; 15–24 = moderate disability; 25–34 = severe disability; over 34 = complete disability [31]. The Spanish validated version was used in this study [32].

#### Quality of life

The Spanish version of the 36-Item Short-Form Health Survey was used to measure quality of life [33]. This survey contains eight dimensions: physical functioning, role limitations due to physical health problems, bodily pain, general health perceptions, vitality, social functioning, role limitations due to emotional problems, and general mental health. The eight dimensions can be summarized in two main components: physical components Short-Form Health Survey-36 (fSF-36) and psychosocial components Short-Form Health Survey-36 (pSF-36) [34]. Scores range from zero (worst health status) to 100 (best health status) [35].

#### Kinesiophobia

The Spanish version of the Tampa Scale for Kinesiophobia (TSK-11) was used to measure the degree of kinesiophobia. The scale consists of 11 questions with four possible answers. The total scale score ranges from 11 to 44, where 11 means no kinesiophobia and 44 means severe kinesiophobia [36, 37].

### Statistical analysis

The statistical analysis was carried out according to the intention to treat principle: all patients, including withdrawals from treatment and patients with poor compliance, remained in the group to which they were assigned by randomisation. Missing values were filled by estimating their values using Multiple Imputation by linear regression for a continuous variable. Besides this, we present a per-protocol analysis which is restricted to a group of patients who have completed the treatment plan and followed the trial protocol instructions exactly. The mean and standard deviations were used as descriptive statistics. The Kolmogorov-Smirnov test was used to verify the normal distribution of the residuals, and Levene’s test confirmed homogeneity. The analysis of covariance (ANCOVA) tested the treatment effect of time (baseline and post- observation assessments) * group (CG vs. EG) for the variations of VAS, NDI, fSF-36, pSF-36 and TSK-11. The effect size of the ANCOVA was calculated with the partial Eta-squared, defined as: 0.01 is small, 0.06 is medium, and 0.14 is large [38]. Additionally, a t-test for independent samples was performed for raw change scores between difference in change between groups for all the analysed variables. Therefore, we used 95% confidence intervals [lower bound, upper bound]. Point estimates on outcomes are presented as original units and standardized effect size were calculated as the between-group difference in means divided by the pooled standard deviation, using the Hedges’ g corrected effect sizes. Hedges’ g were interpreted using the following cut-off values: 0 to 0.2: very small; from 0.2 to 0.5: small; from 0.5 to 0.8: moderate; and from 0.8: strong [38]. The significance level was set at p < 0.05. All analyses were performed using Stata 16.0 for MacOS® software (Stata Corporation, College Station, TX, USA).

## Results

**Fig. 1:**
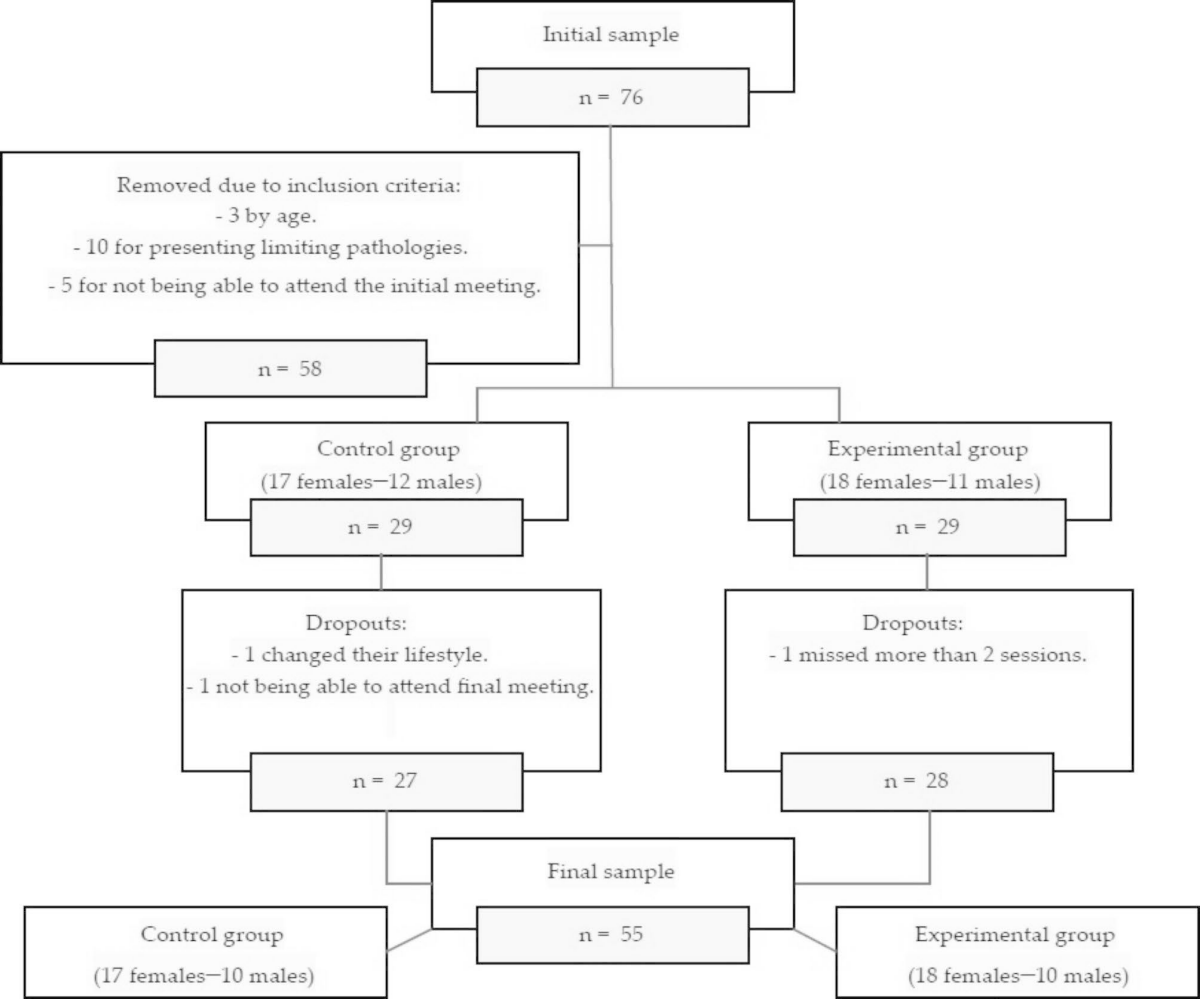
Sample selection flowchart

The sample, after applying the inclusion and exclusion criteria, consisted of a total of 58 participants. During the study, there were three dropouts: two belonging to the CG and one belonging to the EG. The final number of participants was 55 (35 women and 20 men) (Fig. 1). The power analysis (1 – β err prob) of the final sample (n = 58) was calculated post-hoc, obtaining 0.9 for p < 0.05 [27].

Table 2 shows the pre-intervention values. None of the participants had any adverse effects.

**Table 2:** Baseline of the studied variables

Variable	All (n = 58)		CG (n = 29)		EG (n = 29)	
	Mean ± SD	P50	Mean ± SD	P50	Mean ± SD	P50
Age (Years)	50.9 ± 8.8	52.5	50.7 ± 10	54	51 ± 7.6	51
Weight (Kg)	62.4 ± 9.4	59	62.2 ± 8.6	59	62 ± 10.1	58.5
Height (cm)	164.8 ± 10.2	160.5	164.8 ± 9.2	162	164.3 ± 11.2	160
BMI (Kg/m^2^)	22.9 ± 1.2	23	22.8 ± 1.2	20	22.8 ± 1	22.9
VAS (mm)	49.8 ± 10.1	50	49.7 ± 10.7	50	50 ± 9.7	50
NDI	11 ± 4.6	11	10.8 ± 5	11	11.1 ± 4.3	11
fSF-36	40.4 ± 6.5	40.5	40.6 ± 6.1	40.4	40.2 ± 7	42.3
pSF-36	56.8 ± 3.6	57.6	57 ± 3.9	57.7	56.6 ± 3.4	57.3
TSK-11	27.8 ± 4.8	27	27.7 ± 5.1	27	27.9 ± 4.7	28

CG: Control group; EG: Experimental group; SD: Standard deviation; P50: Median; BMI: Body mass index; VAS: Visual Analogue Scale; NDI: Neck Disability Index; fSF-36: physical components Short-Form Health Survey-36; pSF-36: psychosocial components Short-Form Health Survey-36; TSK-11: Tampa Scale of Kinesiophobia.

These results refer to the intention-to-treat analysis (Table 3). The ANCOVA revealed significant treatment effect on VAS (Model constant coefficient 52.42; CI95% [ 49 to 55.8], *F* = 150.95; p < 0.001; η_p_^2^ = 0.73), NDI (Model constant coefficient 11.91; CI95% [10.6 to 13.2], *F* = 45.26; p < 0.001; η_p_^2^ = 0.44), fSF-36 (Model constant coefficient 39.69; CI95% [37.7 to 41.6], *F* = 4.41; p = 0.01; η_p_^2^ = 0.07), and TSK-11 (Model constant coefficient 29.7; CI95% [28.1 to 31.3], *F* = 35.8; p < 0.001; η_p_^2^ = 0.39), while no significant treatment effect was found on pSF-36 (Model constant coefficient 57.04; CI95% [55.9 to 58.2], *F* = 0.25; p = 0.7; η_p_^2^ = 0.004). Additionally, a t-test for independent samples revealed differences in change between both groups. The t-test revealed significant treatment effect on VAS (-11 points; CI95% [5.6 to 16.6], *t* = 4.02, *p* < 0.001, g = 1.04), NDI (-4 points, CI95% [2.5 to 6.2], *t* = 4.74, *p* < 0.001, g = 1.23), fSF-36 (3 points, CI95% [-4.4 to -2.5], *t* = -7.25, *p* = 0.01, g = -1.88), and TSK-11 ( 7 points, CI95%[-8.3 to -5.4], *t* = 7.85, *p* < 0.001, g = 2.04), while no significant differences were found on pSF-36 (-0.02, CI95% [-1.7 to 1.8], *t* = 0.03, g = 0.01, *p* = 0.98).

**Table 3:** Inferential statistics of the ANCOVA test per intention to treat

Variable	Group (N)	Pre-test Mean ± SD	Post-test Mean ± SD	Mean differences [95% CI]	p value	Hedges’ g
VAS (mm)	CG (29)	49.7 ± 10.7	21 ± 13	-29 [-34 to -24]	p < 0.001	1.04
	EG (29)	50 ± 9.7	10 ± 7.3	-40 [-42 to -37]		
NDI	CG (29)	10.8 ± 5	5.9 ± 4.6	-5 [-6.1 to -3.8]	p < 0.001	1.23
	EG (29)	11.1 ± 4.3	1.8 ± 0.9	-9.3 [-10.8 to -7.8]		
fSF-36	CG (29)	40.6 ± 6.1	41.9 ± 5	1.4 [0.7 to 2]	p = 0.01	-1.88
	EG (29)	40.2 ± 7	45 ± 6.1	4.8 [4.1 to 5.5]		
pSF-36	CG (29)	57 ± 3.9	57.2 ± 3.6	0.2 [-0.7 to 1.1]	p = 0.7	0.01
	EG (29)	56.6 ± 3.4	56.8 ± 3.9	0.2 [-1.4 to 1.7]		
TSK-11	CG (29)	27.7 ± 5.1	24.9 ± 5.5	-2.8 [-4.4 to -1.3]	p < 0.001	2.04
	EG (29)	27.9 ± 4.7	17 ± 2.4	-10.8 [-12.4 to -9.3]		

N: sample; SD: standard deviation; CI: Confidence Interval; CG: control group; EG: experimental group; VAS: Visual Analogue Scale; p: p value; NDI: Neck Disability Index; fSF-36: physical Short-Form Health Survey-36; pSF-36: psychosocial Short-Form Health Survey-36; TSK-11: Tampa Scale of Kinesiophobia.

At post-test, the VAS score improved by 80% in the EG and a 58% in the CG, the NDI score improved by 84% in the EG and a 45% in the CG, the fSF-36 score improved by 12% in the EG and a 3% in the CG, the pSF-36 score improved a 0.4% in both groups, and TSK-11 score improved a 39% in the EG and a 10% in the CG (Table 3).

The per-protocol analysis was restricted to 55 patients: 28 in the EG and 27 in the CG. These patients were excluded if they missed more than two BSP sessions in the EG, one changed his lifestyle and other did not be able to attend final meeting. The results of the per protocol analysis (Table 4) were similar to the results of the intention to treat analysis (Table 3): both analyses showed significant differences in the VAS, NDI, fSF-36 and TSK-11, and neither showed significant differences in the pSF-36.

**Table 4:** Inferential statistics of the ANCOVA test per protocol

Variable	Group (N)	Pre-test Mean ± SD	Post-test Mean ± SD	Mean differences [95% CI]	p value	Hedges’ g
VAS (mm)	CG (27)	50 ± 11	21 ± 13	-29 [-34 to -24]	p < 0.001	0.99
	EG (28)	50 ± 10	10 ± 7	-40 [-42 to -37]		
NDI	CG (27)	10.9 ± 5	5.9 ± 4.6	-5 [-6.1 to -3.8]	p < 0.001	1.2
	EG (28)	11.1 ± 4.3	1.8 ± 0.9	-9.3 [-10.8 to -7.8]		
fSF-36	CG (27)	40.6 ± 6.3	41.9 ± 5.2	1.3 [0.6 to 2.1]	p = 0.021	-1.84
	EG (28)	40.2 ± 7.1	44.9 ± 6.2	4.8 [4.1 to 5.6]		
pSF-36	CG (27)	57 ± 4	57.2 ± 3.7	0.2 [-0.8 to 1.2]	p = 0.814	0.02
	EG (28)	56.6 ± 3.5	56.8 ± 3.9	0.2 [-1.5 to 1.8]		
TSK-11	CG (27)	27.7 ± 5.3	24.9 ± 5.7	-2.9 [-4.4 to -1.3]	p < 0.001	2
	EG (28)	27.9 ± 4.8	17 ± 2.5	-10.8 [-12.4 to -9.3]		

N: sample; SD: standard deviation; CI: Confidence Interval; CG: control group; EG: experimental group; VAS: Visual Analogue Scale; p: p value; NDI: Neck Disability Index; fSF-36: physical Short-Form Health Survey-36; pSF-36: psychosocial Short-Form Health Survey-36; TSK-11: Tampa Scale of Kinesiophobia.

**Fig. 2:**
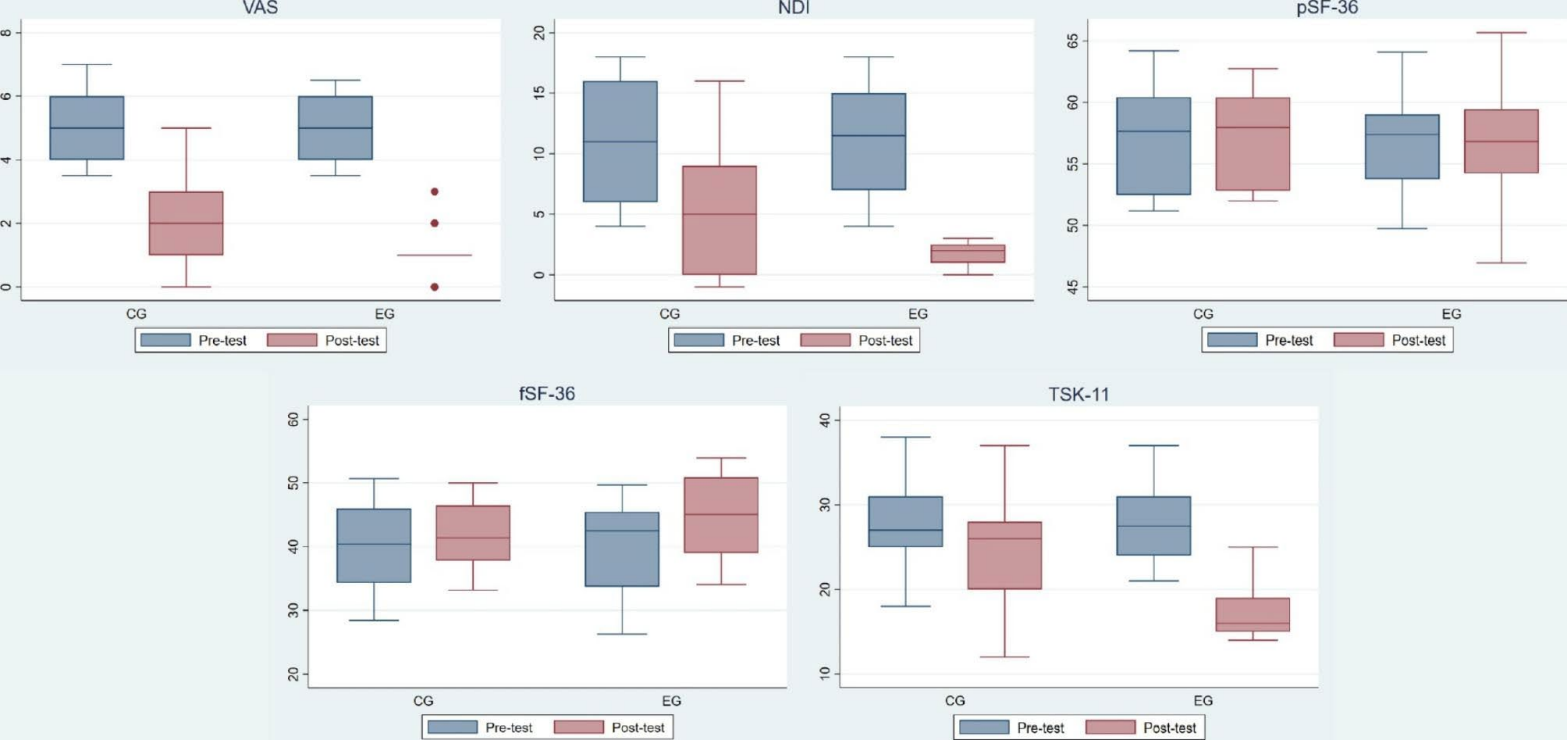
Box and whisker plot for the outcomes

Figure 2 shows graphically the results of the variables VAS, NDI, fSF-36, pSF-36 and TSK-11, in the CG and EG, before and after the intervention.

## Discussion

The aim of this research was to determine the effects of a BSP-based intervention for the treatment of patients with NNP in an adult population. The results of the study suggest that the effects are positive, including those obtained in pain reduction and improvement of disability and kinesiophobia.

Participants who performed BSP showed minimal clinically important differences in pain intensity as defined by Kovacs et al. [39], with a score improvement of 40 points on the VAS (however, the authors are aware that this type of results should be analyzed with caution [40, 41]). Although it is worth mentioning that the measurement tool used in the study by Kovacs et al. [39] was The Numerical Rating Scale. The VAS and The Numerical Rating Scale are very similar scales for measuring pain [42], so as there are no studies that calculate the minimal clinically important differences with the VAS, we have used the study by Kovacs et al. [39] as a reference. This finding is congruent with the beneficial effects of BSP-based interventions on non-specific low back pain [21–26]. This may be due to the fact that many risk factors for non-specific back pain are common to both regions, for example: biological factors (loss of muscle strength and motor control), psychological factors (stress or anxiety) or social factors (catastrophic view of pain and incorrect social beliefs) [11]. This BSP intervention follows the recommendations of the biopsychosocial model of pain [43], thus taking into account all these risk factors. These risk factors are taken into account, since, in the practical part, the patients perform motor control and strength exercises, and, in the theoretical part, they are aware of the erroneous catastrophic beliefs regarding the causes and origin of back pain [19]. Different studies also show improvements in neck pain through exercise or health education [44, 45]. However, it should be mentioned that, with education alone, the effects on pain are small and could be insufficient as the only treatment for patients with non-specific spinal pain [46]. The improvements obtained in the CG (in addition to being non-significant) were less than four points and can therefore be considered clinically irrelevant [39].

In parallel, the identified benefits on disability have been clinically relevant according to the definition of Young et al. [47]. They set the minimal clinically important differences at 7.5 points difference in the NDI [47]. However, the CG only improved by five points. These results are consistent, since disability is strongly related to pain, due to the close relationship between the physical and psychosocial components [48]. In the same line, other authors who applied BSP in the lumbar region also obtained positive results [21–27], which could be due to the fact that many risk factors of lumbar pain are common to the risk factors of the cervical region [11]. A recent review concludes that exercise therapy produces an improvement in function in the cervical region [49]. This review highlights that this improvement increases in multimodal interventions and that interventions with a duration between 6 and 12 weeks are more effective, both of which are characteristics of this BSP intervention [49].

Changes in quality of life were clinically relevant for fSF-36 but not for pSF-36 [50]. Although previous research has demonstrated the benefits of BSP on quality of life in both components of the SF-36 [23, 26]. These findings could be due to the fact that the baseline pSF-36 scores were 12.3% higher than the mean scores in the Spanish population [35]. Consequently, having such high scores before the start of the study may have made it difficult to obtain meaningful results on this variable.

The BSP intervention resulted in beneficial changes in kinesiophobia with a strong effect size. These results are in agreement with other studies in which exercise and education were combined in the treatment of NNP [51, 52]. The observed improvement in kinesiophobia could be due to the fact that exercise therapy and health education are fundamental for its treatment [53]. The International Association for the Study of Pain also establishes a relationship between fear-pain-knowledge, since they state that pain represents not only the sensation of physical harm, but also an emotional experience that can be influenced by other emotions, such as anxiety or fear of the unknown [54]. For all these reasons, the biopsychosocial approach is the current paradigm in the treatment of non-specific back pain [17]. Furthermore, disability is also related to kinesiophobia [55]. This relationship may confirm the benefits found in both variables in this study.

As limitations to the study, it should be mentioned that our study did not have a post-intervention follow-up. Another major limitation is that only 24-hour acute pain was assessed and not the characteristics of chronic (or longer-term) pain. In addition, although VAS is widely used to assess pain intensity in clinical and epidemiological settings, measurement of pain intensity by VAS is influenced by subjective pain perception [56]. Finally, the limited number of participants prevented stratification of results by age and gender. For future research, it would be interesting to include long-term post-intervention follow-ups and to include larger sample sizes that allow stratification of the results.

There is evidence about the benefits of BSP in the treatment of low back pain [21–26] and, in view of the results obtained on the different variables in this study, it seems that BSP can also help treating NNP. Along the same lines, a review of Clinical Practice Guidelines on the treatment of neck pain highlights the importance of exercise therapy and health education for the good prognosis of the patient with NNP [57].

## Conclusions

The BSP-based theoretical and practical programme had beneficial effects on pain in patients with NNP. In addition, this programme reduced disability, kinesiophobia in NNP patients and improved the physical component of quality of life. However, the psychosocial component of quality of life did not change after participation in the BSP.

This programme could be implemented in physiotherapy clinics, primary care centers or hospitals, reducing the severe socio-economic impact caused by NNP.

## Electronic supplementary material

Below is the link to the electronic supplementary material.

Supplementary Material 1

## Data Availability

The datasets used and/or analyzed during the current study are available from the corresponding author on reasonable request.

## List of Abbreviations

BSP: Back School Program
fSF-36: Physical components Short-Form Health Survey-36
NDI: Neck Disability Index
NNP: Non-specific neck pain
pSF-36: Psychosocial components Short-Form Health Survey-36
TSK-11: Tampa Scale for Kinesiophobia
VAS: Visual Analogue Scale
