# Supplementary material for: Effects of back school-based intervention on non-specific neck pain in adults: a randomized controlled trial
Source: BMC Sports Sci Med Rehabil. 2023 Apr 17;15:60. doi: 10.1186/s13102-023-00666-8 (PMC10111684; doi:10.1186/s13102-023-00666-8)
Supplement: Supplementary file 1 — Supplementary Material 1 [file 13102_2023_666_MOESM1_ESM.docx]

| **Appendix 1. Main part of the practical intervention.** | | | | |
| --- | --- | --- | --- | --- |
| Name | Starting position | End position | Active rest | Duration |
| Isometric cervical extensions | 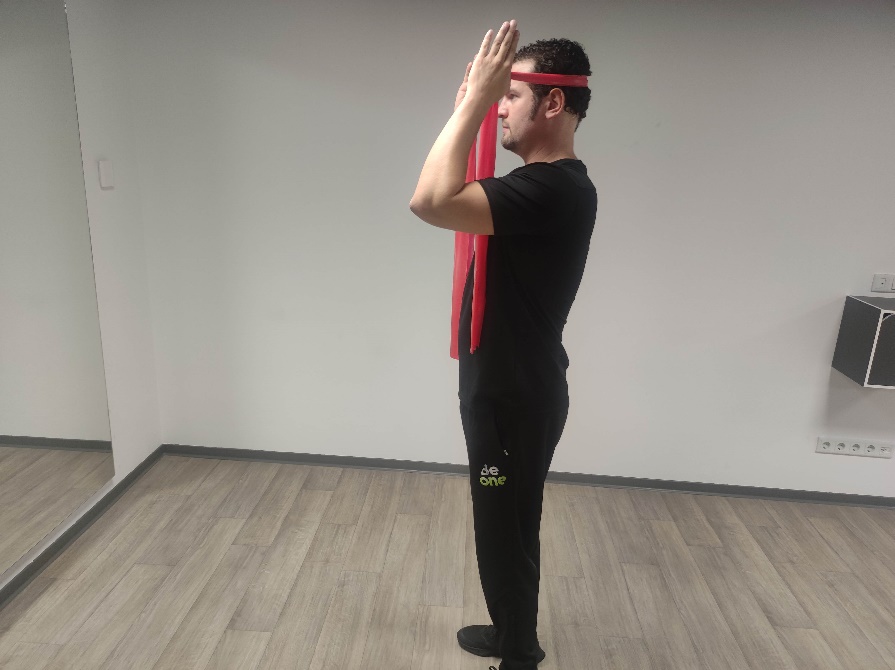 | 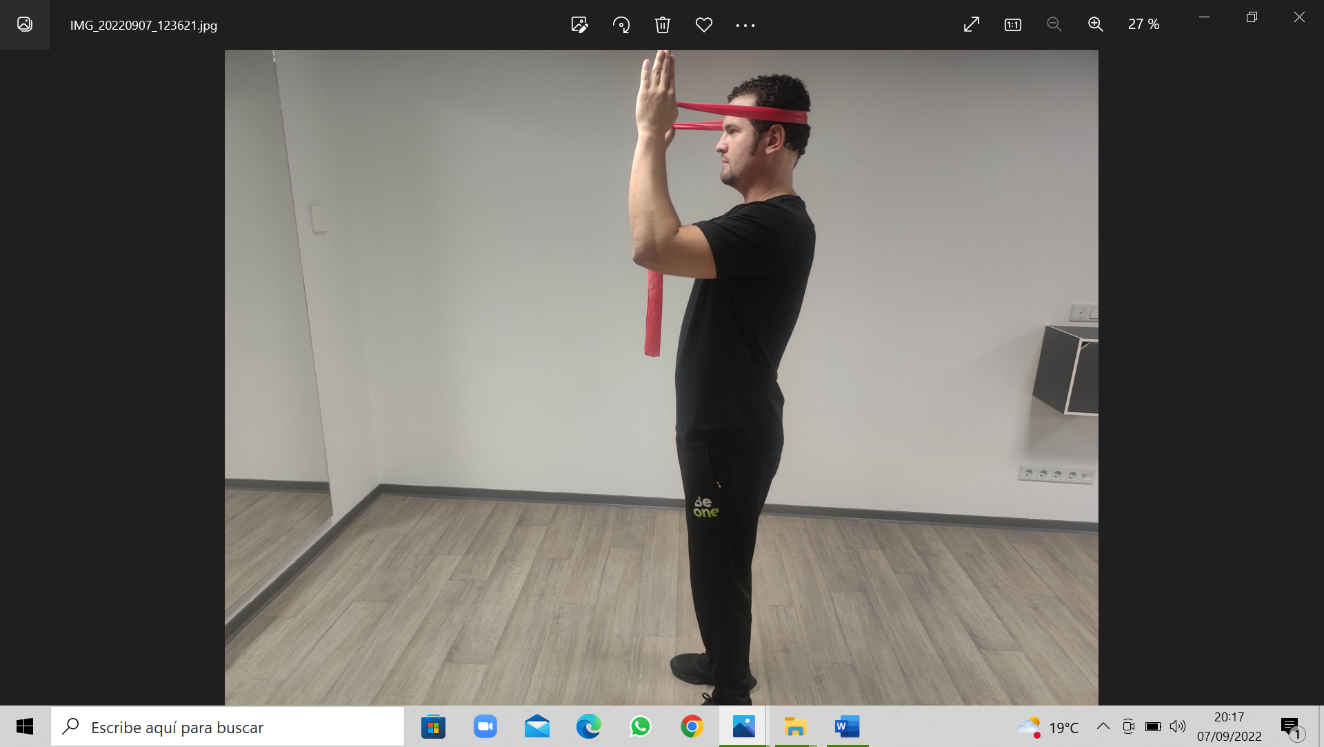 | Neck flexion-extension without resistance | Exercise  3x30´´  Rest  3x10´´ |
| Right isometric cervical tilts | 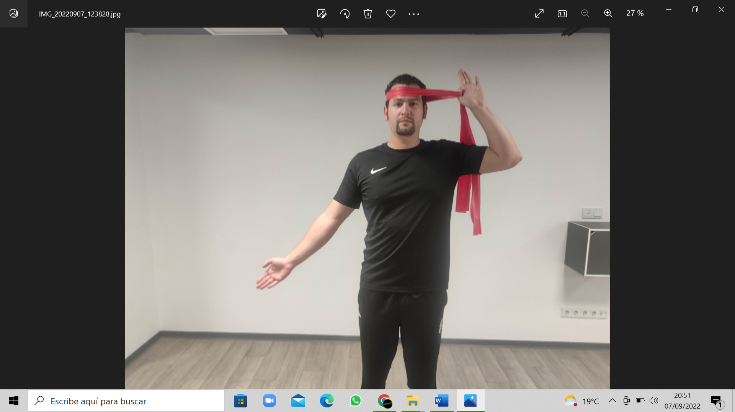 | 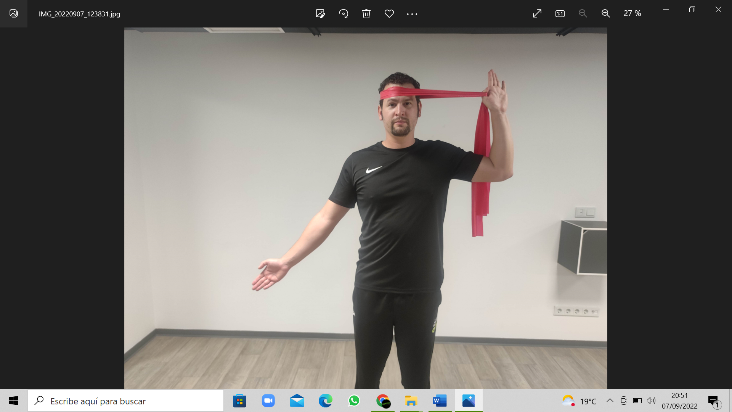 | Neck tilts from right to left | Exercise  3x30´´  Rest  3x10´´ |
| Left isometric cervical tilts | 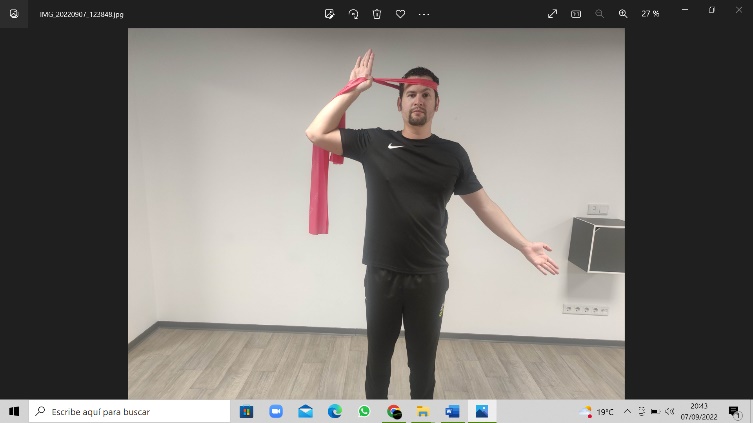 | 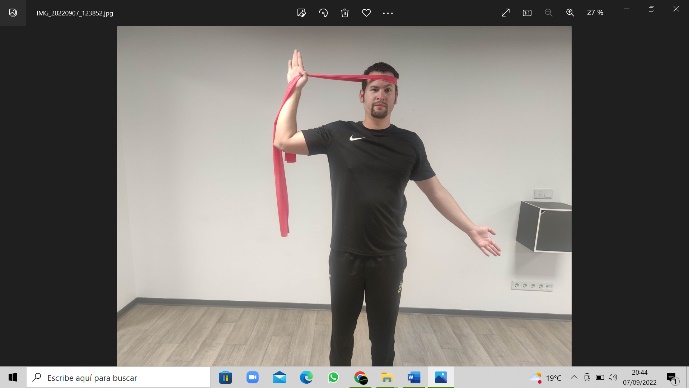 | Neck tilts from right to left | Exercise  3x30´´  Rest  3x10´´ |
| Isometric cervical flexions | 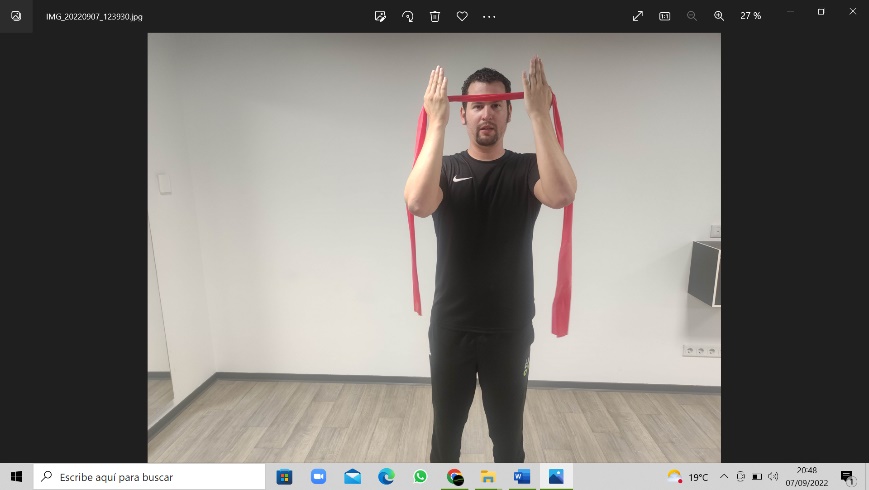 | 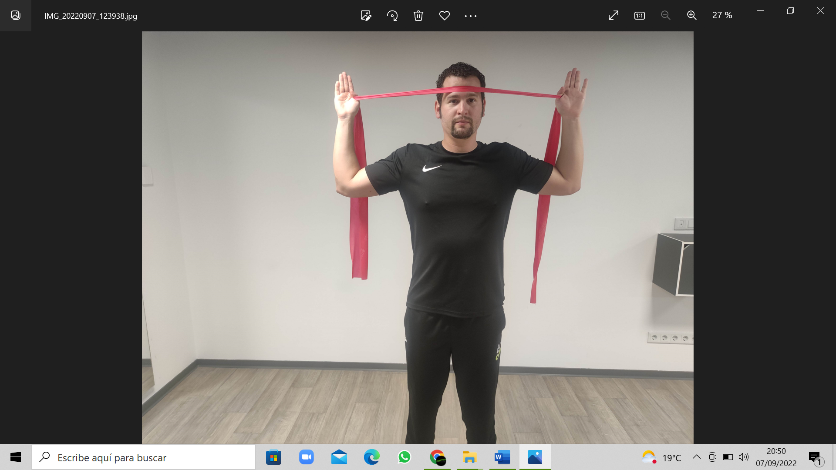 | Neck flexion-extension without resistance | Exercise  3x30´´  Rest  3x10´´ |
| Right shoulder external rotations | 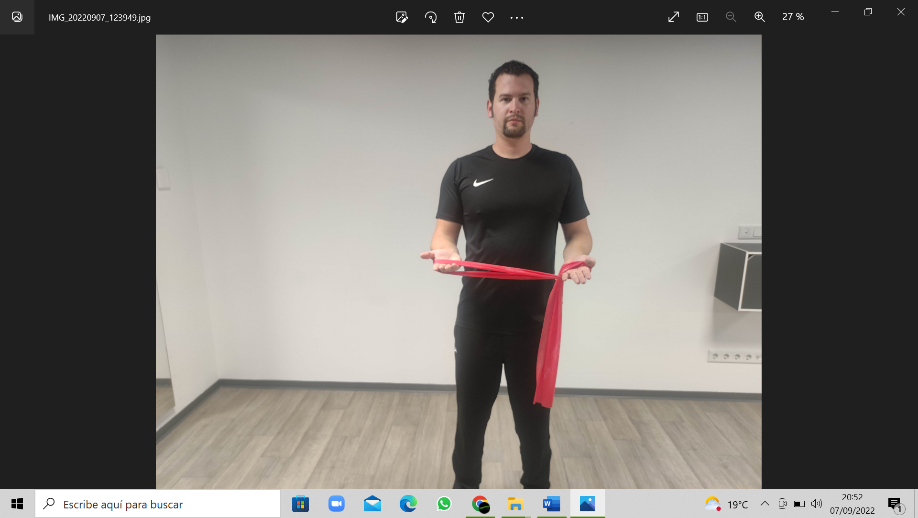 | 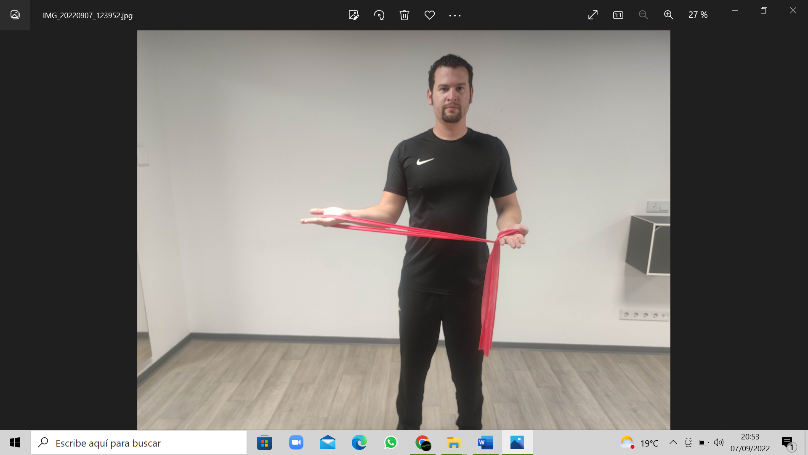 | Back shoulder circles | Exercise  3x30´´  Rest  3x10´´ |
| Left shoulder external rotations | 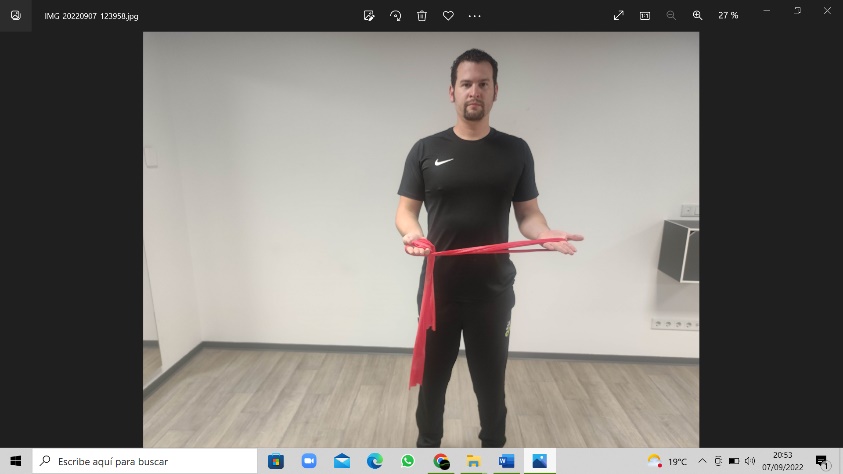 | 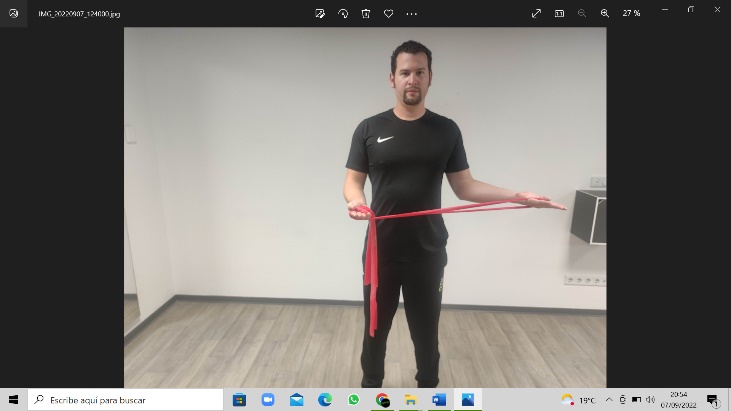 | Posterior arm rotation | Exercise  3x30´´  Rest  3x10´´ |
| Scapular stabilizations | 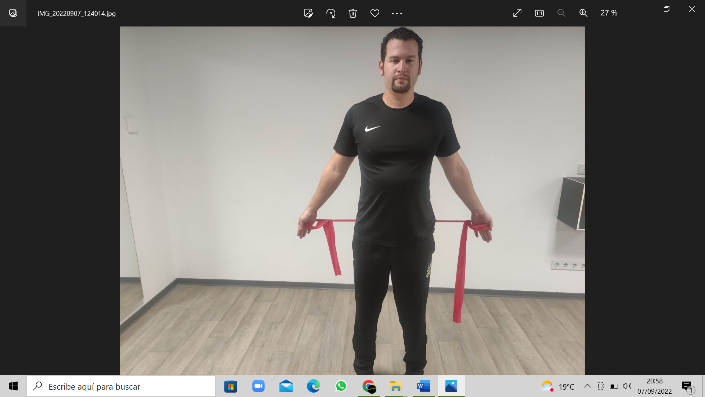 | 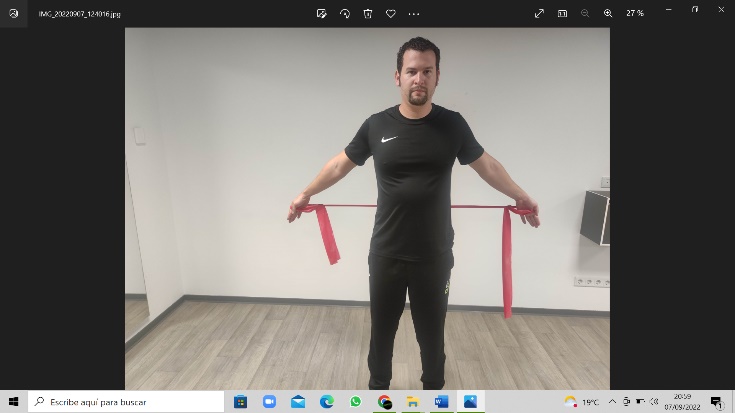 | Posterior arm rotation | Exercise  3x30´´  Rest  3x10´´ |
| **Appendix 1. Main part of the practical intervention *(cont.)*** | | | | |
| Name | Starting position | End position | Active rest | Duration |
| Postero-anterior oscillations | 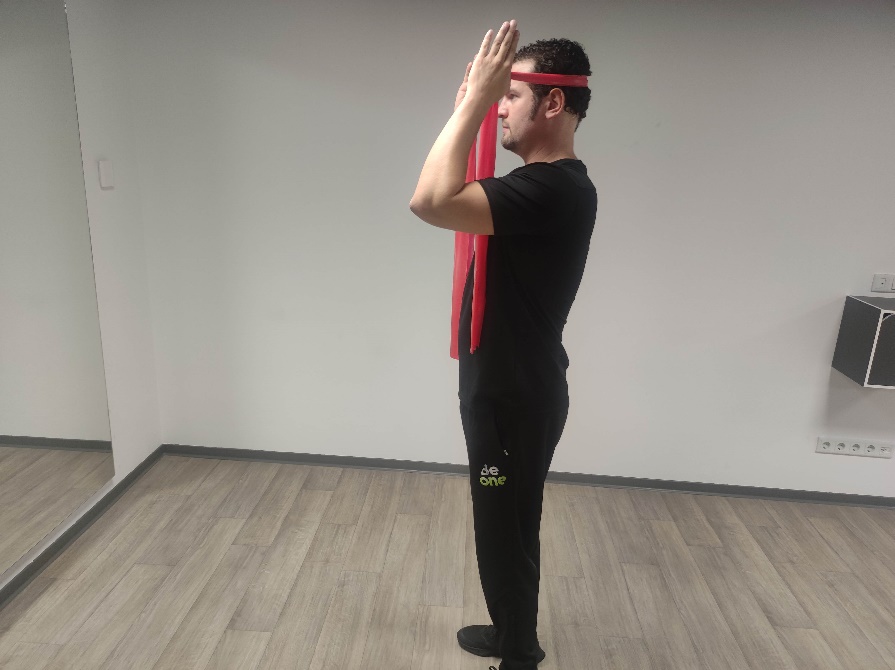 | 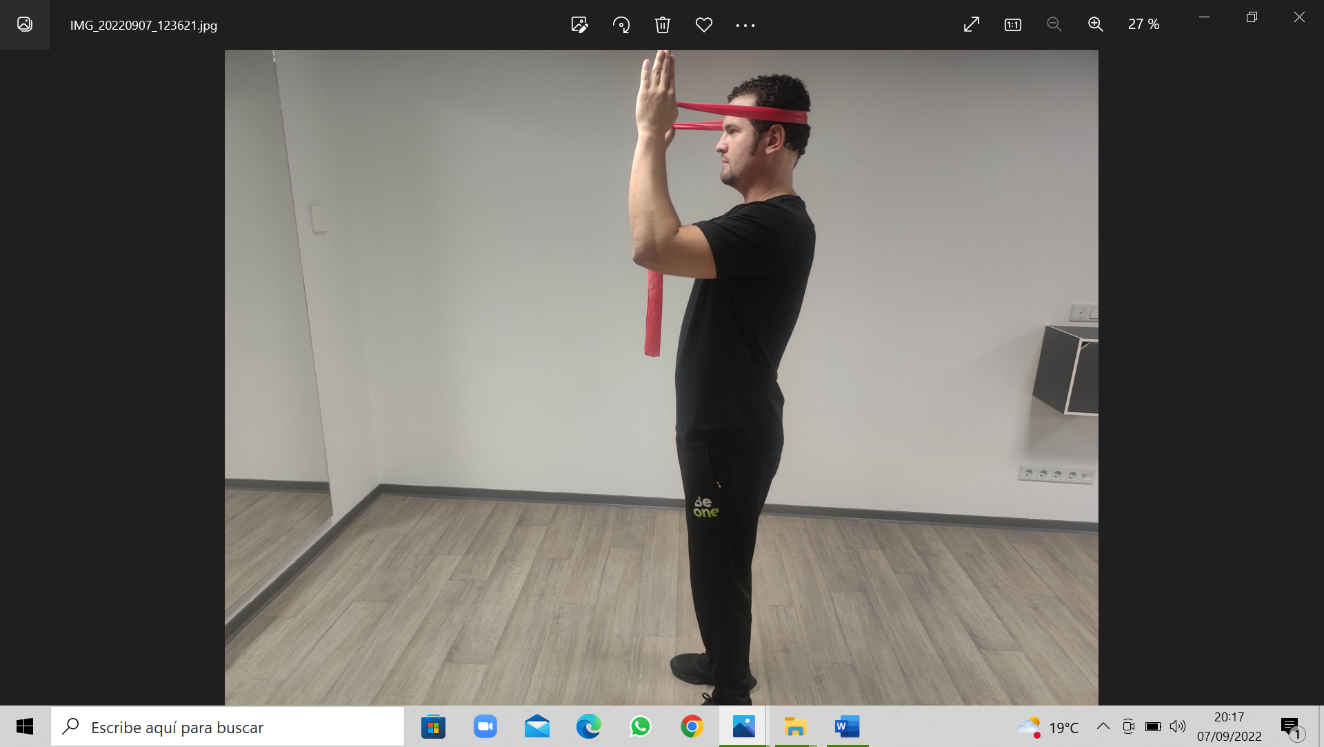 | Neck flexion-extension without resistance | Exercise  3x30´´  Rest  3x10´´ |
| Right lateral oscillations | 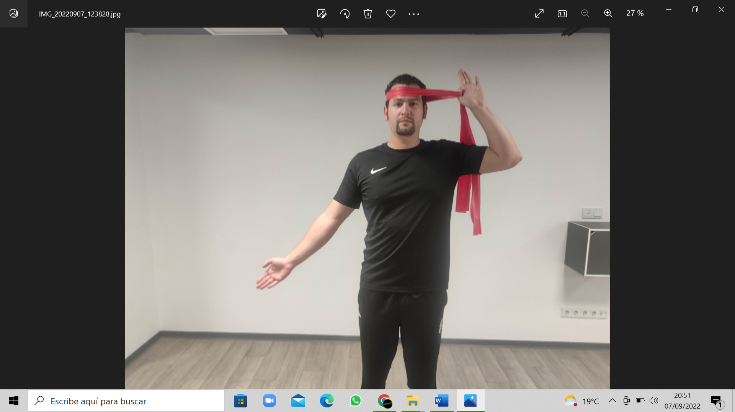 | 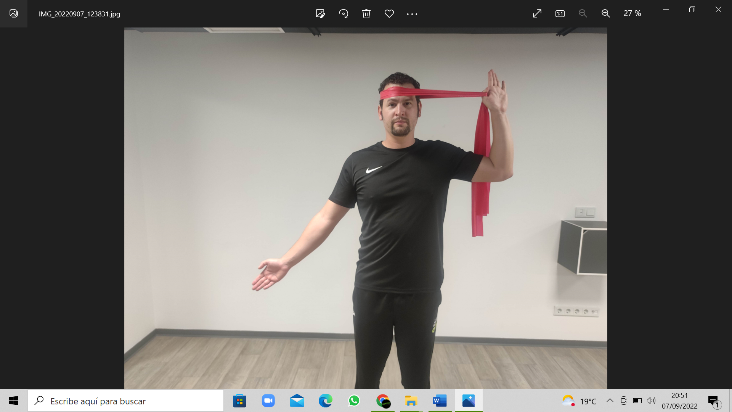 | Neck tilts from right to left | Exercise  3x30´´  Rest  3x10´´ |
| Left lateral oscillations | 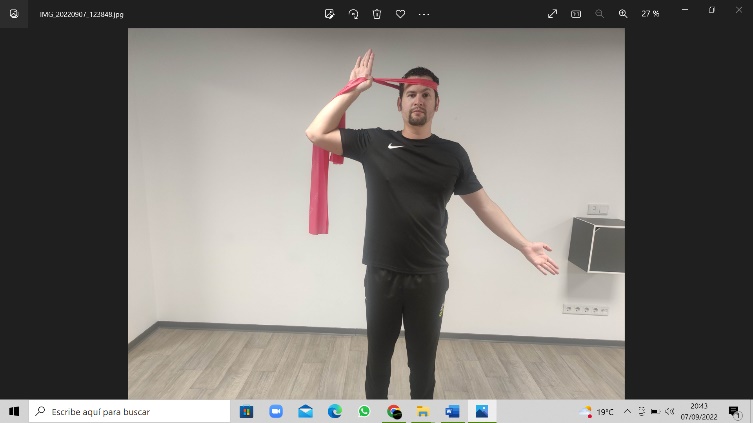 | 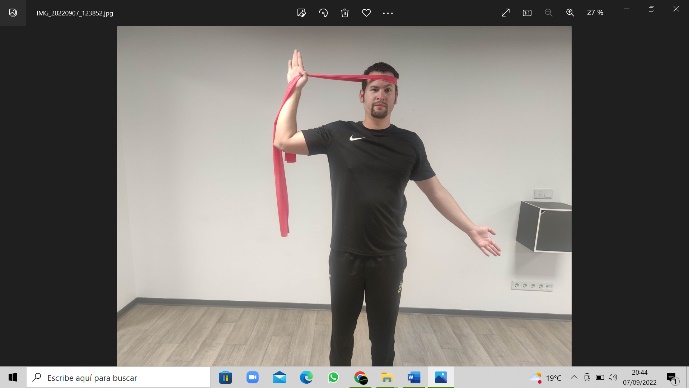 | Neck tilts from right to left | Exercise  3x30´´  Rest  3x10´´ |
| Antero-posterior oscillations | 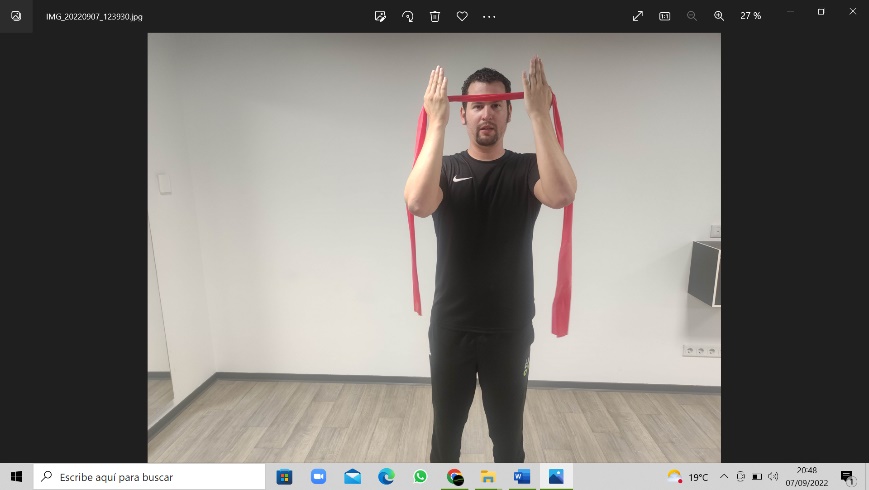 | 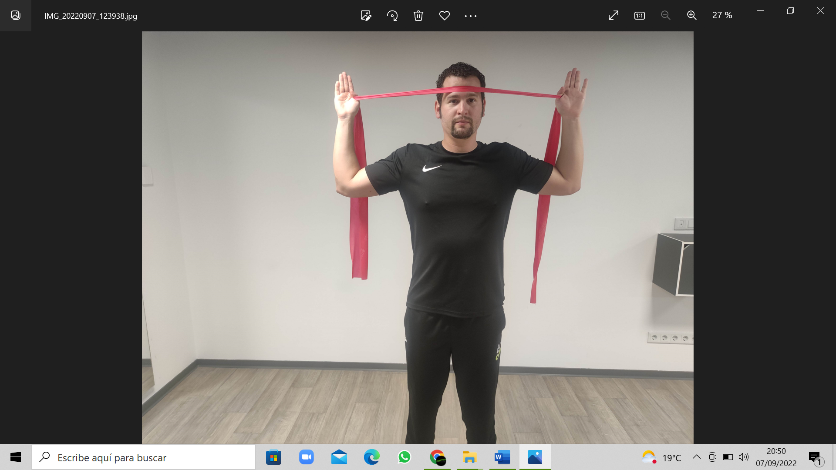 | Neck flexion-extension without resistance | Exercise  3x30´´  Rest  3x10´´ |
| Prone cervical extensions | 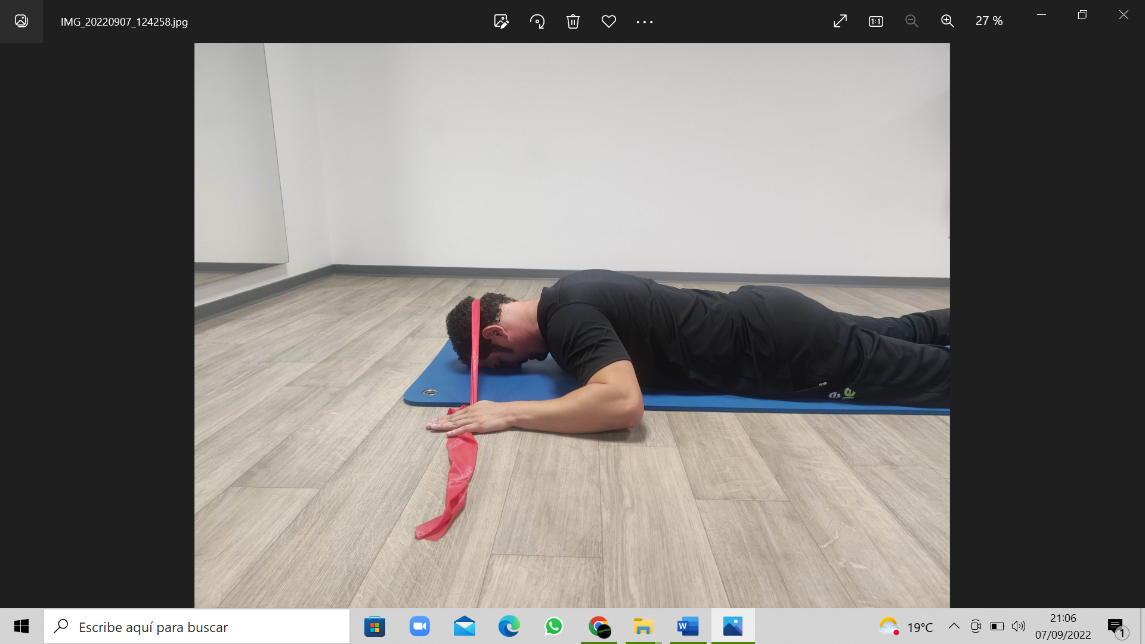 | 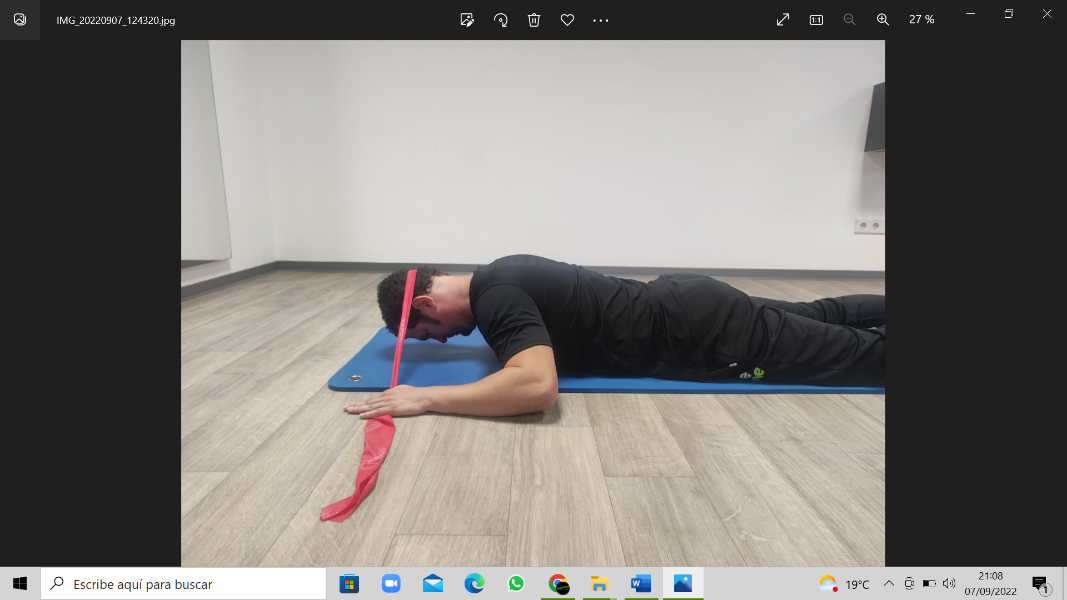 | Back shoulder circles | Exercise  3x30´´  Rest  3x10´´ |
| Prone scapula stabilizations | 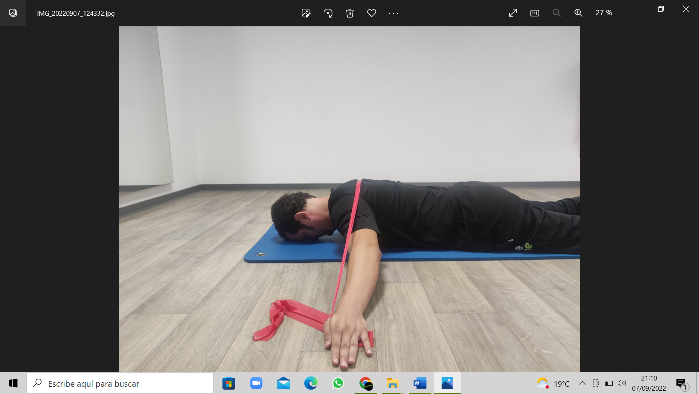 | 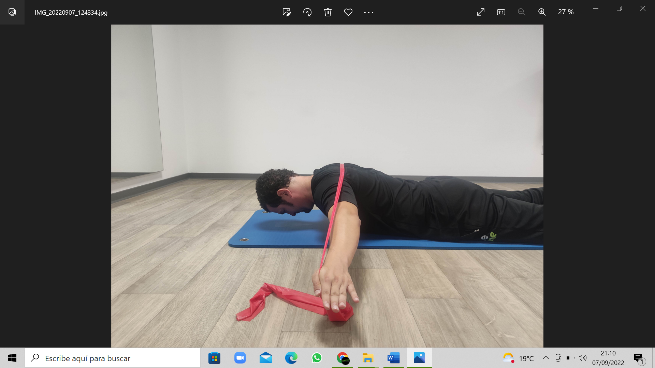 | Posterior shoulder circles | Exercise  3x30´´  Rest  3x10´´ |
| Supine cervical flexions | 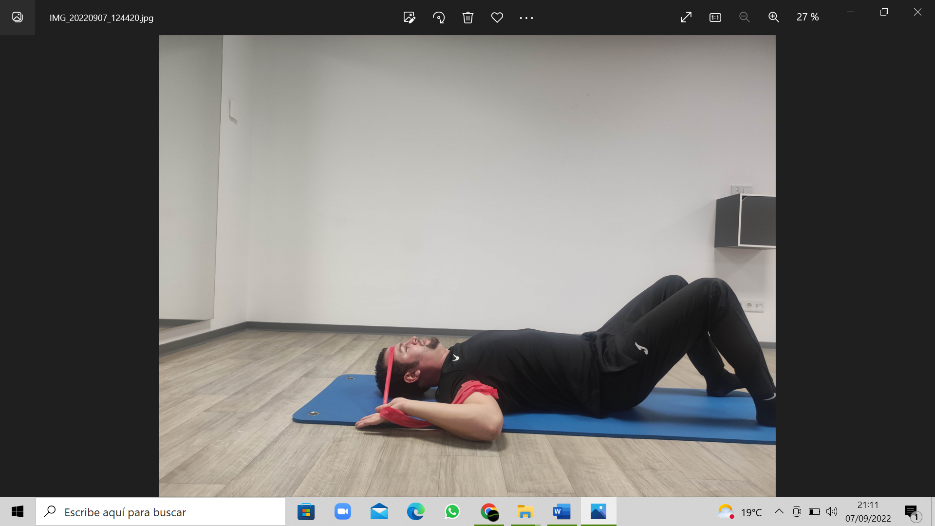 | 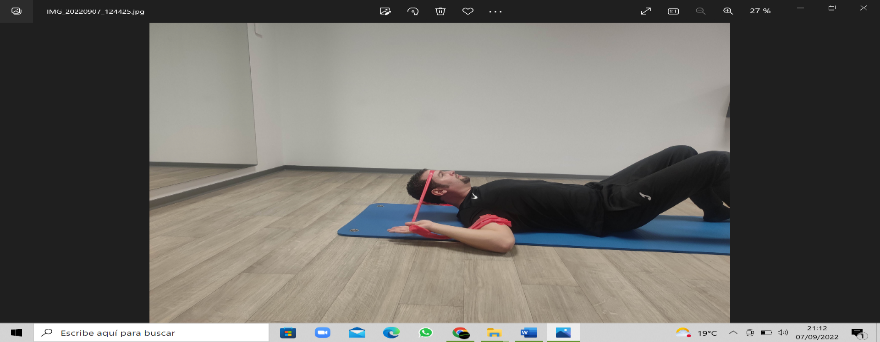 | Back shoulder circles | Exercise  3x30´´  Rest  3x10´´ |
| Supine shoulder external rotations | 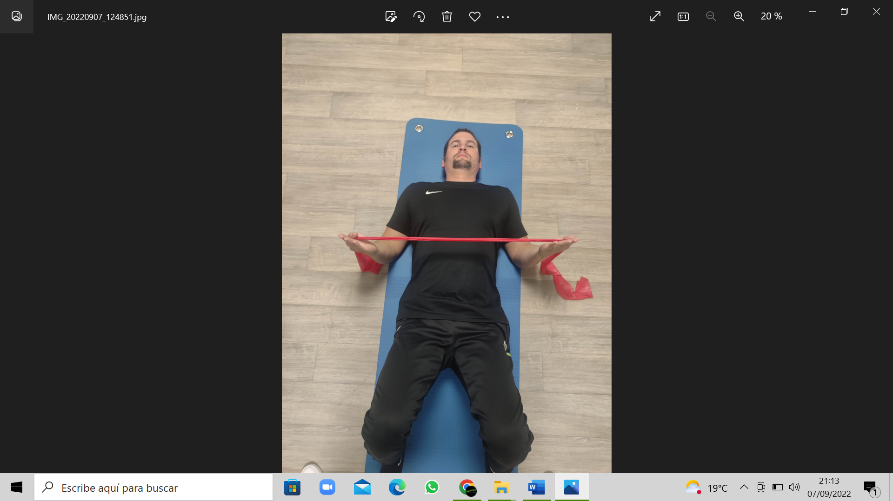 | 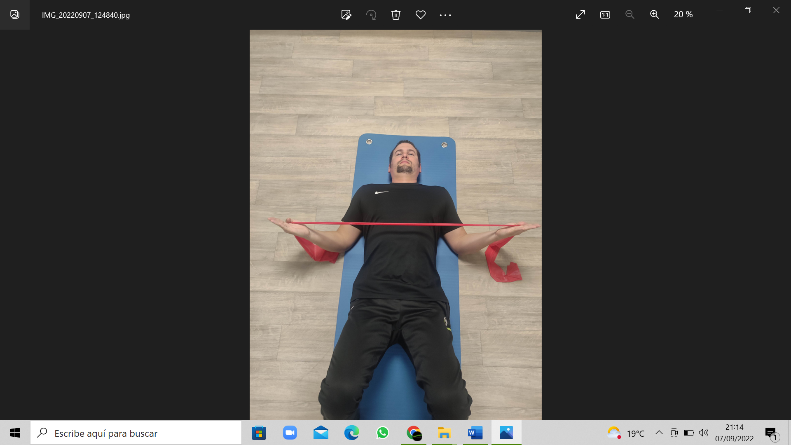 | Posterior shoulder circles | Exercise  3x30´´  Rest  3x10´´ |
